# Proactive Engagement of the Expert Meeting in Managing the Early Phase of the COVID-19 Epidemic, Japan, February–June 2020

**DOI:** 10.3201/eid2710.204685

**Published:** 2021-10

**Authors:** Tomoya Saito, Kaori Muto, Mikihito Tanaka, Nobuhiko Okabe, Hitoshi Oshitani, Satoshi Kamayachi, Yoshihiro Kawaoka, Akihiko Kawana, Motoi Suzuki, Kazuhiro Tateda, Hitomi Nakayama, Masaki Yoshida, Akifumi Imamura, Fumio Ohtake, Norio Ohmagari, Ken Osaka, Mitsuo Kaku, Tomimasa Sunagawa, Kazutoshi Nakashima, Hiroshi Nishiura, Koji Wada, Shigeru Omi, Takaji Wakita

**Affiliations:** National Institute of Infectious Diseases, Tokyo, Japan (T. Saito, M. Suzuki, T. Sunagawa, T. Wakita);; Institute of Medical Science, The University of Tokyo, Tokyo (K. Muto, Y. Kawaoka);; Waseda University Graduate School of Political Science, Tokyo (M. Tanaka);; Kawasaki City Institute for Public Health, Kawasaki, Japan (N. Okabe);; Tohoku University Graduate School of Medicine, Sendai, Japan (H. Oshitani); Japan Medical Association, Tokyo (S. Kamayachi);; University of Wisconsin–Madison, Madison, Wisconsin, USA (Y. Kawaoka);; National Defense Medical College, Tokorozawa, Japan (A. Kawana);; Toho University School of Medicine, Tokyo (K. Tateda);; Kasumigaseki–Sogo Law Office, Tokyo (H. Nakayama);; Jikei University School of Medicine, Tokyo (M. Yoshida);; Tokyo Metropolitan Cancer and Infectious Diseases Center Komagome Hospital, Tokyo (A. Imamura);; Osaka University Graduate School of Economics, Toyonaka, Japan (F. Ohtake);; National Center for Global Health and Medicine, Tokyo (N. Ohmagari);; Tohoku University Graduate School of Dentistry, Sendai (K. Osaka);; Tohoku Medical and Pharmaceutical University, Sendai (M. Kaku);; Daito Bunka University, Higashimatsuyama, Japan (K. Nakashima);; Graduate School of Medicine and Faculty of Medicine Kyoto University, Kyoto, Japan (H. Nishiura);; International University of Health and Welfare, Narita, Japan (K. Wada);; Japan Community Health Care Organization, Tokyo (S. Omi)

**Keywords:** policy making, communicable diseases, health communication, risk assessment, public health practice, COVID-19, coronavirus disease, SARS-CoV-2, severe acute respiratory syndrome coronavirus 2, viruses, respiratory infections, zoonoses, Japan

## Abstract

To deal with the risk of emerging diseases with many unknowns, close and timely collaboration and communication between science experts and policymakers are crucial to developing and implementing an effective science-based intervention strategy. The Expert Meeting, an ad hoc medical advisory body, was established in February 2020 to advise Japan’s COVID-19 Response Headquarters. The group played an important role in the policymaking process, promoting timely situation awareness and developing science-based proposals on interventions that were promptly reflected in government actions. However, this expert group may have been overly proactive in taking on the government’s role in crisis management. For the next stage of managing the coronavirus disease pandemic and future pandemics, the respective roles of the government and its advisory bodies need to be clearly defined. Leadership and strategic risk communication by the government are key.

The coronavirus disease (COVID-19) pandemic has had a huge global impact. Historically, the role of the scientific community has been crucial for developing science-based interventions to be implemented by public health authorities seeking to effectively confront newly emerging infectious diseases. In Japan’s first-phase response to the COVID-19 pandemic, which lasted through the end of June 2020, the Expert Meeting (EM) for COVID-19 response served as an advisory group for the government’s COVID-19 Response Headquarters. The group played a much more extensive role than groups of technical experts are generally expected to play. This article reviews the literature on the role and achievements of the EM in the COVID-19 response during February–June 2020, with the aim of defining the ideal relationship and mechanism of expert groups and government authorities for future public health crisis management.

## Operational and Advisory Bodies for COVID-19 Response in Japan

At the end of December 2019, when cases of human infection by the new coronavirus were first reported, the Government of Japan (GOJ) began to carefully monitor the epidemic situation in China and raise awareness among local governments and medical doctors to promote early detection (*1*). The Ministry of Health, Labour and Welfare (MHLW) established the MHLW COVID-19 Response Headquarters on January 28, 2020. In response to the declaration of Public Health Emergency of International Concern issued by the World Health Organization on January 30, 2020 (*2*), GOJ established the ad hoc GOJ COVID-19 Response Headquarters (ad hoc GOJ HQ) to coordinate multisector collaboration. The MHLW response HQ established its Advisory Board in early February to initially provide expert input regarding the COVID-19 outbreak aboard the Diamond Princess cruise ship, which was docked in Japan. After 2 meetings (Table 1; Figure), the EM was established in the ad hoc GOJ HQ on February 14, 2020. The EM’s mandate was to give expert medical advice pertaining to COVID-19 countermeasures (*3*). All members of the Advisory Board were assigned to the newly established GOJ EM, and further meetings of the MHLW Advisory Board were suspended.

**Table 1:** Agenda of the Advisory Board for the Minster of Health, Labour, and Welfare regarding COVID-19 in Japan, February 2020

Meeting no.	Date	Agenda
1	Feb 7	Intervention strategy for a cruise ship outbreak, testing policy for asymptomatic cases, infectivity and pathogenicity of SARS-CoV-2
2	Feb 10	Intervention strategy for a cruise ship outbreak, testing policy for detecting cases, infectivity and virulence of COVID-19, infectivity of asymptomatic cases, risk for virus mutation

*COVID-19, coronavirus disease; SARS-CoV-2, severe acute respiratory syndrome coronavirus 2.

**Figure:**
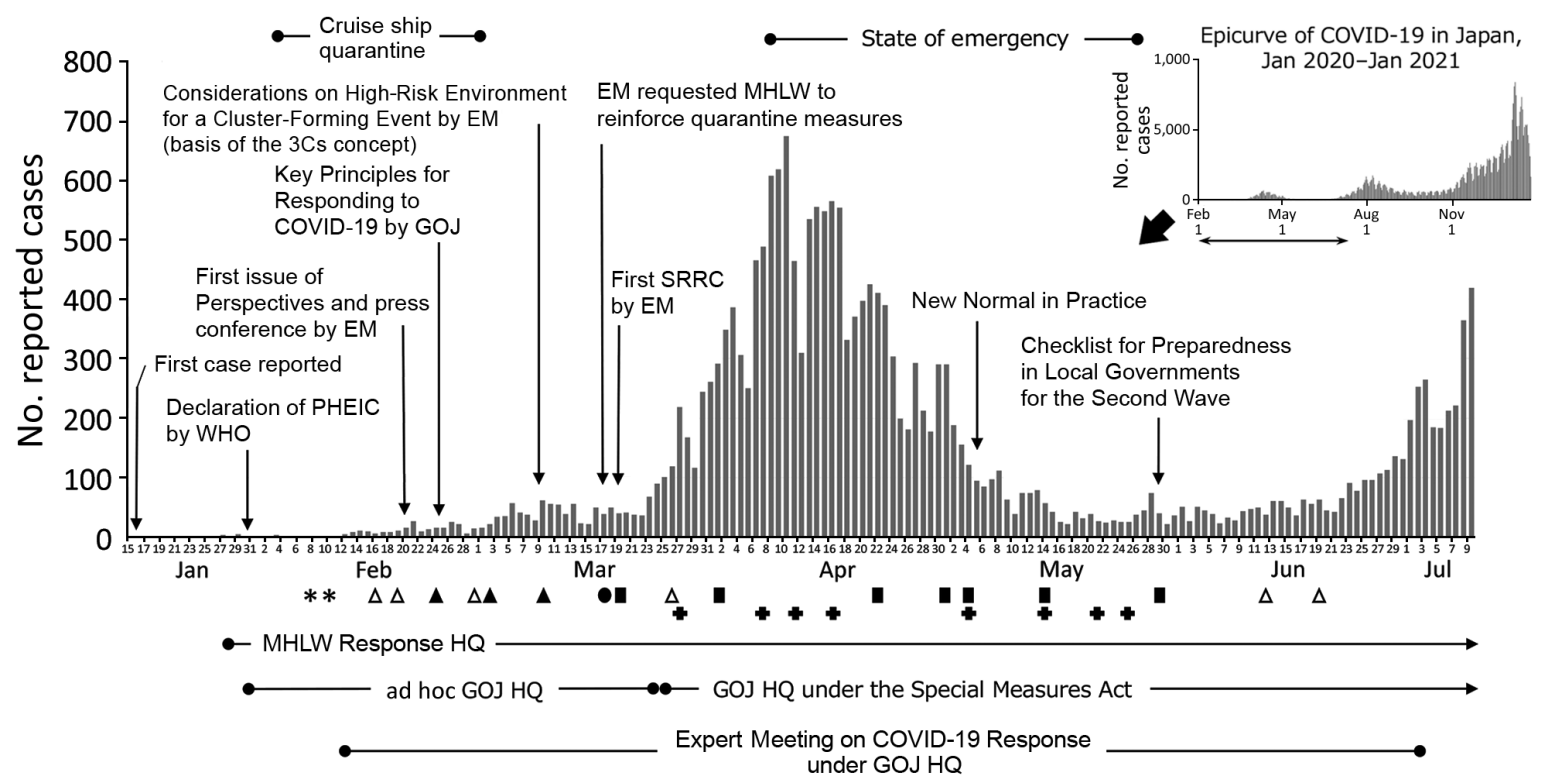
Major activities of the EM and epidemiologic curve of (COVID-19 in Japan, January–July 2020. The GOJ ad hoc GOJ HQ on January 30, 2020, as an ad hoc response headquarters to respond to the COVID-19 epidemic with the Cabinet’s approval. The Advisory Board in the MHLW COVID-19 Response Headquarters was established on February 4 and had several meetings (indicated by asterisks). However, all members were assigned to a newly established advisory body, the EM in the ad hoc GOJ HQ, on February 14, 2020, which actively discussed and proposed COVID-19 response measures to ad hoc GOJ HQ until July 3, 2020. White triangles, black triangles, black circles, and black squares all indicate days when the EM was held. Black triangles indicate when EM published issues of its Perspective. Black circles indicate when EM published its Request to the MHLW on quarantine measures. Black squares indicate when EM published its Situation Report and Recommendations on COVID-19 Epidemic. Meetings of the Advisory Board were not held during the time the EM was active. After the Special Measures Act for Pandemic Influenza and New Infectious Disease Preparedness and Response was amended to apply this act to COVID-19, the GOJ COVID-19 Response HQ (GOJ HQ) was established under the Special Measures Act on March 26 when COVID-19 was recognized as having pandemic potential. This new GOJ HQ took over the role of coordinating the comprehensive government response to COVID-19 from its predecessor, the ad hoc GOJ HQ. The Advisory Committee on Basic Policies is a standing subcommittee of the Panel of Experts for Pandemic Influenza and New Infectious Diseases mandated to give advice on the basic policies for responding to the pandemic under the Special Measures Act. The meetings of the Advisory Committee on Basic Policies were held upon the publication and amendment of the Basic Policies beginning on March 27, 2020 (indicated by black plus signs). 3Cs, closed spaces with poor ventilation, crowded places with many persons nearby, and close-contact settings such as close-range conversations; COVID-19, coronavirus disease; EM, Expert Meeting; GOJ, Government of Japan; HQ, headquarters; MHLW, Ministry of Health, Labour and Welfare; MHLW Response HQ, MHLW COVID-19 Response Headquarters; PHEIC, Public Health Emergency of International Concern; SRRC, Situation Report and Recommendations on COVID-19 Epidemic; WHO, World Health Organization.

The EM was composed of 10 members with backgrounds in pediatrics, internal medicine, respiratory medicine, epidemiology, clinical infectious disease, infection control, virology, public health, practice law, and medical sociology. These members were selected by the ad hoc GOJ headquarters (*3*); most had previous experience with pandemic preparedness and emergency response. The president of the National Institute of Infectious Diseases chaired the EM. Other experts were invited to participate in the meetings as needed, including those with expertise in mathematical modeling and environmental health. In addition to holding formal meetings, EM members would frequently engage unofficially in person or by video conference.

On March 26, GOJ transitioned the ad hoc GOJ HQ to an official GOJ Headquarters (GOJ HQ) to play a coordinating role under the Special Measures Act for Pandemic Influenza and New Infectious Diseases Preparedness and Response (Special Measures Act). The Advisory Committee on Basic Policies (BP Advisory Committee) is a standing subcommittee of the Panel of Experts for Pandemic Influenza and New Infectious Diseases, mandated to give advice on “basic policies” for responding to the pandemic under Article 18 of the Special Measures Act and to provide guidance on appropriate countermeasures during the response. In addition to the assigned members before the emergence of severe acute respiratory syndrome coronavirus 2, all the EM members were included in the BP Advisory Committee, as suggested by Dr. Shigeru Omi, the vice-chair of the EM and the chair of the BP Advisory Committee. Meetings of the BP Advisory Committee were held after the publication and amendment of the Basic Policies on March 27, 2020; however, the EM continued to hold official meetings and play its role as an advisory body through the end of June (Figure).

## Activities of the Advisory Bodies in Early February

The initial roles of the Advisory Board in the MHLW and the EM in the ad hoc GOJ HQ in early February were limited to expressing their opinions on items included in the meeting agenda set by the government; however, their comments were immediately reflected in government policy (Tables 1, 2). For example, the government’s discharge policy for asymptomatic patients was modified to use negative test results on February 18 (*19*), soon after a discussion of the issue by the EM on February 16 (*20*). The group’s discussion of mass gathering events on February 19 (*21*) was quickly followed by the Health Minister’s alert, issued on February 20 (*4*), and the Prime Minister’s request on February 26, that persons refrain from hosting mass gathering events (*22*). Public health measures (e.g., surveillance and prevention of the spread of infection) and the capacity of the medical system to prepare for the pandemic phase were discussed at the fourth (*23*) and fifth meetings of the EM (*24*), subsequent to which notifications were sent to local governments to enhance their preparedness for the pandemic phase on March 1 (*6*) and March 6 (*8*).

**Table 2:** Agenda and outcomes of the Expert Meeting for COVID-19, Japan, 2020

Meeting no.	Date	Major agenda	Related actions
1	Feb 16	Characteristics of COVID-19, situational assessment, patient visitation numbers, isolation of asymptomatic cases	–
2	Feb 19	Situational assessment, policies on mass gathering events, passenger management policy of a cruise ship	Alert regarding hosting mass gathering events from MHLW on Feb 20 (*4*)
3	Feb 24	Key principles for responding to COVID-19	Published first issue of Perspectives (*5*), press conference
4†	Feb 29	Preparedness for public health measures (surveillance, prevention of the spread, medical preparedness) in a pandemic phase	Notified local governments to enhance preparedness on Mar 1 (*6*).
5†	Mar 2	Pandemic scenario and medical preparedness, countermeasures for outbreaks in Hokkaido Prefecture	Published Perspectives on COVID-19 Epidemic (*7*), notified local governments to enhance medical preparedness on Mar 6 (*8*).
6	Mar 9	Epidemiologic assessment, evaluation of interventions in Hokkaido Prefecture, considerations on the high-risk environment for a cluster-forming event in daily life	Published Perspectives on COVID-19 Epidemic (*7*), press conference, publication of Considerations on the High-Risk Environment for a Cluster-Forming Event (*9*)
7†	Mar 17	Risk for imported cases, quarantine measures	Published Request to MHLW on quarantine measures (*10*), quarantine measures reinforced and the area of refusal of landing expanded on Mar 18
8	Mar 19	Epidemiologic assessment, behavior changes to avoid the 3Cs, medical preparedness, stigma and discrimination, diagnostics, policy on mass gathering events, consultation guidance on COVID-19 for patients	Published first Situation Report and Recommendations on COVID-19 Epidemic (*11*)
9†	Mar 26	Epidemiologic assessment	–
10	Apr 1	Situation report and recommendations, standards for discharge for mild cases	Published Situation Report and Recommendations on COVID-19 Epidemic (*12*), press conference
11	Apr 22	Situation report and recommendations, hospitalization policy for pregnant women	Published Situation Report and Recommendations on COVID-19 Epidemic (*13*), press conference
12	May 1	Situation report and recommendations	Published Situation Report and Recommendations on COVID-19 Epidemic (*14*), press conference
13	May 4	Situation report and recommendations, consultation guidance for COVID-19 for patients	Published Situation Report and Recommendations on COVID-19 Epidemic (*15*), press conference, published a supplemental document on surge capacity assessment on molecular diagnostics (*16*), published New Normal in Practice (*16*)
14	May 14	Situation report and recommendations	Published Situation Report and Recommendations on COVID-19 Epidemic (*17*) and press conference
15	May 29	Situation report and recommendations, review of discharge policy, review of testing policy for close contacts	Published Situation Report and Recommendations on COVID-19 Epidemic (*9*), press conference, published Checklist for Preparedness in Local Governments for the Second wave (*18*), published supplemental document on Japan’s cluster-based approach (*10*)
16†	June 12	Revision of discharge policy	–
17†	June 19	Revised pandemic scenario and medical preparedness	–

*3Cs, closed spaces with poor ventilation, crowded places with many persons nearby, and close-contact settings such as close-range conversations; COVID-19, coronavirus disease; –, no specific action taken. †Round-robin.

## Expert Meeting’s Proactive Engagement in Developing Intervention Strategies Such as 3Cs

During the first 2 weeks of February, MHLW concentrated on the missions of 4 chartered flights to evacuate Japanese citizens from China’s Wuhan Province and on the safe disembarkation of passengers and crew from the quarantined Diamond Princess cruise ship. Recognizing the potential risk and impact of delay, and the changing situation of the COVID-19 pandemic nationwide, members of the EM saw the need for immediate precautionary actions and assumed a more active role. The members agreed to propose measures proactively to GOJ rather than merely responding passively to the government’s agenda. After the group’s third meeting (*25*), the Health Minister accepted the group’s proposal that the EM publish “Perspectives,” enabling the EM to communicate its opinions and recommendations directly to citizens.

The EM’s first issue of Perspectives was published on February 24 (*5*), 1 day before publication of the GOJ Key Principles Responding to COVID-19 (*26*). The initial Perspectives characterized as a high-risk environment for COVID-19 transmission any occasion “where people meet many others face-to-face at close range and have a conversation for a certain period.” The publication expressed concern regarding the chain of events where 1 infected person infects many others and thus forms a cluster. This concern became the basis for Japan’s “cluster-based approach” to COVID-19 (*10*,*27*). The EM hosted a press conference on the day of publication and described the epidemiologic situation as “critical” over the coming 1–2 weeks.

After the release of GOJ Key Principles on February 25 (*26*), GOJ intensified its response to COVID-19. On February 26 (*22*), the Prime Minister asked the public not to host any mass-gathering event for the next 2 weeks. He also asked that schools be closed for the entire term until the scheduled spring break (*28*), a measure he took without consulting with the EM.

In response to the spread of COVID-19 in Hokkaido Prefecture, where the governor had declared a nonbinding state of emergency on February 28, the EM provided an epidemiologic assessment and proposed necessary measures for the prefecture (*7*). The EM called for action by the residents and businesses of Hokkaido to reduce human-to-human contact and avoid high-risk environments. The EM also issued an alert regarding the potential risk of younger people being spreaders.

Perspectives proposed the 3 pillars of the COVID-19 response strategy: early detection and response to clusters, having the medical capacity for early diagnosis and intensive care for severely ill patients, and self-motivated behavior changes by citizens (*9*). The EM also published “Considerations Regarding a High-Risk Environment for a Cluster-Forming Event in Daily Life” (Considerations) (*9*). Considerations provided critical information for preventing the formation of clusters, recommending the avoidance of the 3Cs (closed spaces with poor ventilation, crowded places with many persons nearby, and close-contact settings such as close-range conversations).

## Publication of “Situation Report and Recommendations”

On March 17, the EM submitted a request to the MHLW to reinforce quarantine measures in response to the increase in imported cases (*29*). After the seventh meeting on March 19, the EM began to publish “Situation Report and Recommendations on the COVID-19 Epidemic (SRRC),” which described measures more comprehensively and covered nonmedical issues, including ethical, legal, and social aspects (*11*). The EM pointed out the risk for a rampant surge of patients as observed in other countries and the possible future need for intensive interventions such as city lockdowns. The group’s recommendations, including cautions regarding prejudice and discrimination against infected persons, their close contacts, and healthcare professionals handling the infection, were directed at the government as well as the country’s citizens and businesses. EM members and responsible officers in the MHLW and the Cabinet Secretariat jointly drafted the report, which was discussed intensively at the meeting and subsequently announced at a follow-up press conference.

## Expert Meeting’s Activities after Activation of the Special Measures Act

The Special Measures Act was amended to apply to the COVID-19 response on March 6. The EM agreed with the situation assessment by the MHLW, pointing out the risk of the COVID-19 pandemic in Japan (*30*). The assessment was then submitted to the Prime Minister. The Prime Minister decided to establish a new GOJ COVID-19 Response Headquarters (GOJ HQ) under Article 15 of the Special Measures Act, thereby formalizing the response framework of its predecessor ad hoc headquarters. Measures such as restricting the use of large facilities to prevent persons from congregating became implementable under the Special Measures Act as part of the response to the epidemic.

At this time, GOJ HQ began to publish its “Basic Policies for Novel Coronavirus Disease Control” (Basic Policies) under the Special Measures Act as a means for taking unified action throughout the country (*31*). Even after this formal framework was activated and roles related to decision making were formally transferred to the BP Advisory Committee, the EM (which was inviting still more stakeholders to attend its sessions, including representatives from local health departments and an expert in behavioral economics) continued to lead the development of policy by proposing concepts supported by extensive situation reports and risk assessments. Because the GOJ included all the members of the EM in the BP Advisory Committee to maintain the consistency of the discussion, EM’s ideas were fully incorporated into Basic Policies, which guided the coordinated national response under the Special Measures Act. This close collaboration may have strengthened the credibility of EM’s technical messages.

On April 1, SRRC proposed indicators for local epidemiologic assessment, medical preparedness, and necessary measures according to the extent of the epidemic at the local level (*12*). It emphasized the urgent need for developing medical surge capacity, especially for severe cases in major cities.

## Proposing the “New Normal” during the State Of Emergency

On April 7, GOJ declared a 1-month state of emergency in 7 prefectures for the COVID-19 outbreak under Article 31 of the Special Measures Act (*32*). The state of emergency was expanded to cover the entire country on April 16 (*33*).

On April 22, SRRC reported its interim analysis of public behavior and pointed out the need for an 80% reduction in human-to-human contact throughout the society of Japan (*13*). The EM expressed concerns regarding the spread of the disease attributable to travel during the national holiday season beginning at the end of April. SRRC also encouraged the securing of more medical and public health capacity under the governors’ leadership. On May 1, SRRC updated its interim analysis of public behavior and proposed the continuation of the state of emergency. It recommended that strict behavioral changes be called for in highly infected areas, and that in areas where the incidence of the disease was limited, residents should be encouraged to lead their lives under a “new normal” (*16*).

On May 4, SRRC updated its situational assessment and reconfirmed the need for a state of emergency (*16*). The EM encouraged the installation of the new normal both in daily life and in business and published the “New Normal in Practice” in an Annex of SRRC (*16*). The EM also proposed considerations for developing infection control guidelines according to the type of business to encourage implementation of infection control in business areas and the restart of social activities (*16*). On the same day, GOJ extended the state of emergency through the end of May (*15*), with consideration given to the epidemic situation and SRRC.

On May 14, SRRC proposed conditions for lifting and re-declaring the state of emergency as well as a step-by-step reopening strategy (*17*). GOJ lifted the state of emergency for 39 of the 47 prefectures on the same day (*34*) and lifted it entirely on May 25 (*35*).

On May 29, SRRC summarized the interim assessment of the epidemic response since January. It concluded that early detection, a cluster-based strategy, and the state of emergency were the keys to success in suppressing the number of cases and deaths in the early phase. SRRC also pointed out problems in diagnosis, medical capacity, the functioning of public health centers, surveillance, infection prevention and control, and research and development for medical countermeasures, and proposed the “Checklist for Preparedness in Local Government for the Second Wave” (*18*).

## The End of the Expert Meeting

The final 2 EM sessions were held round-robin on June 12 (*36*) and June 19 (*37*). At the respective meetings, participants discussed the revision of the discharge policy and a revised pandemic scenario and medical preparedness, which were passed on to the local governments later the same day (*38*,*39*).

On July 3, the Cabinet dissolved the EM and established as its successor the Subcommittee on COVID-19 Measures under the Ministerial Meeting on Pandemic Influenza and Novel Infectious Disease, a standing advisory body authorized by the Special Measures Act. This subcommittee was tasked with discussing broader issues, including monitoring the COVID-19 epidemic, vaccination policy, and countermeasures for a secondary wave (*40*).

## Discussion

For emerging diseases such as COVID-19, to interpret risk assessment on the basis of limited and incomplete information and implement the most effective measures promptly, close communication and coordination between experts and public health authorities is essential. Although it was an ad hoc entity, the EM played an important role in leading the discussion of countermeasures against a new viral disease with pandemic potential.

Most of the EM’s proposed policy options regarding countermeasures were immediately reflected in the actions of the GOJ. The most important achievements of the EM were the establishment of a cluster-based approach to reducing infections and its proposal of the 3Cs concept for raising public awareness of high-risk environments, a concept that is now widely used in COVID-19 prevention campaigns such as that conducted by the World Health Organization (*41*). Through the EM’s publications and its frequent press conferences, the public was informed in a timely manner and updated with concise and clear messages on the current situation and ways to protect themselves from infection. Throughout every activity on scientific arguments and the advice as to its fruition, the EM tried to make a consensus with thorough deliberation. The messages from the EM were unified in their publications. Members also agreed on maintaining the accessibility to journalists for transparency. Sometimes members commented through mass and online media about their perspective concerning the integrated message from the EM.

In general, the involvement of experts in the policy-making process has been rather passive in Japan. However, the EM was proposing its own agenda, formulating its own views, and holding press conferences to communicate its proposals to the public. This type of proactive involvement in policy-making and communication by an expert body within the government was quite heretical in the history of Japan’s scientific governance; besides the experience of the 2009 influenza A(H1N1) pandemic (*42*,*43*) or the devastating triple disaster (an earthquake, a tsunami, and a nuclear accident at Fukushima) that occurred in 2011, the scientific advisory body was required to be passive and reactive in the governance system. Nevertheless, many experts’ reports of those incidents called for total reform in gathering disciplinary scientists’ knowledge and evidence promptly, giving unerring and continuous scientific advice to the government and establishing the risk communication circuits. In the case of COVID-19, the GOJ still requested passive and reactive roles from only a small number of experts. But EM members decided to suggest policy options proactively to spur immediate countermeasures, with the words, “we have crossed the Rubicon.”

Indeed, the request for financial support from the government for businesses to increase adherence to the government’s request to suspend business activities (*12*) and the EM’s detailed proposal on the ways of the new normal (*16*) may well have gone beyond medical and public health advice. Its detailed messaging on public health interventions through the publication of Perspectives and SRRC, and its frequent press conferences, may have heightened the public perception that the EM played a larger role than it should have. The EM’s approach may have been misperceived by a public that saw that the group was making decisions on any policy (*44*–*46*). However, the EM became proactive in part because of the reluctance of the GOJ to provide adequate risk communication. Risk communication is 1 of the 5 key findings pointed out in the Joint External Evaluation Mission Report in Japan as essential for reinforcing health security (*47*).

In the next stage of the COVID-19 epidemic, and for the future in general, the government must take the lead in risk communication, and the various professional advisory bodies need to play a cooperative role. Intrinsically, an expert advisory body should be responsible for assessing the epidemiologic situation and making recommendations to the government based on its assessment. The government should then decide whether to accept or reject the recommendations and be responsible for implementing and communicating its policies. Furthermore, a strategic plan for risk communication based on public engagement should be established in GOJ. Communication efforts should not be limited to press releases or press briefings. Countermeasures should be designed and proposed to reflect the latest knowledge and to update the risk assessment; they should be communicated carefully to the public, with consideration of the potential impact and damage to the lives of citizens and the risk perception of diverse audiences. The government, risk communication experts, and expert advisory bodies should discuss what information the government should deliver and how it should be delivered. The central government’s official communications with local governments through government notices and memoranda, which play a central role in implementing policies, must be more transparent and articulate. The government should seek out the advice of experts, recognizing that they are an integral part of risk communication. When seeking better scientific advice under science-in-action situations, independent (but not overly competitive) groups consisting of interdisciplinary experts would be required to tame the uncertain situation. When the Advisory Board was established, the MHLW added quasi-experts to the list, such as a law practitioner and a medical sociologist; the selection of such persons subsequently proved advantageous for expanding the expert networks and communicating with the public. This effort cannot be undertaken by the scientific community alone; rather, the government needs to help organize and listen to them to arrive at a better decision.

At the press conference after the 17th meeting of the EM on June 24, the group’s members suggested that GOJ reorganize the advisory body to redefine its role and responsibilities and the role and obligations of the government (*25*). Three proposals were put forward, calling for clear role-sharing between the government and the advisory body, leadership and strategies regarding risk communication, and the promotion of interdisciplinary academic collaboration to include ethical, legal, and social issues. The Cabinet subsequently abolished the EM and established the Subcommittee on COVID-19 Measures under the Panel of Experts for Pandemic Influenza and New Infectious Diseases, a standing advisory body authorized by the Special Measures Act (*40*). On July 14, the Advisory Board to the MHLW was reactivated to specifically address public health issues and situational assessment (*48*). The performance of the new advisory bodies and that of the government, together with their expected roles and responsibilities, will need be reviewed and assessed.

In conclusion, the ad hoc EM proactively engaged in Japan’s early phase COVID-19 response. It was successful during the early phase of the epidemic; however, to promote effective crisis management in the future, the respective roles and shared responsibilities of such expert groups and the government need to be reconsidered. A clear delineation of their roles, together with a systematic and extensive communication of risk by the government, are essential components for effectively combatting COVID-19 in its next phase and for managing any future pandemic.
